# Harvesting the wisdom of the crowd: using online ratings to explore care experiences in regions

**DOI:** 10.1186/s12913-018-3566-z

**Published:** 2018-10-20

**Authors:** Roy J P Hendrikx, Marieke D Spreeuwenberg, Hanneke W Drewes, Jeroen N Struijs, Dirk Ruwaard, Caroline A Baan

**Affiliations:** 1 Tranzo Scientific Center for Care and Welfare, Research Centre for Technology in Care, Tilburg University, PO Box 90153, 5000 LE Tilburg, The Netherlands; 2 Zuyd University of Applied Sciences, PO Box 550, 6400 AN Heerlen, The Netherlands; 3 Department of Health Services Research, Care and Public Health Research Institute (CAPHRI) , Faculty of Health, Medicine and Life Sciences, Maastricht University, PO Box 616, 6200 MD Maastricht, The Netherlands; 4 Department for Quality of Care and Health Economics, Center for Nutrition, Prevention and Health Services, National Institute for Public Health and the Environment, PO Box 1, 3720 BA Bilthoven, The Netherlands; 5 Department of Public Health and Primary Care, LUMC Campus, Schouwburgstraat 2, 2522 VA The Hague, The Netherlands

**Keywords:** Population health management, Regional evaluation, Quality of care, Online ratings, Unsolicited data

## Abstract

**Background:**

Regional population health management (PHM) initiatives need an understanding of regional patient experiences to improve their services. Websites that gather patient ratings have become common and could be a helpful tool in this effort. Therefore, this study explores whether unsolicited online ratings can provide insight into (differences in) patient’s experiences at a (regional) population level.

**Methods:**

Unsolicited online ratings from the Dutch website Zorgkaart Nederland (year = 2008–2017) were used. Patients rated their care providers on six dimensions from 1 to 10 and these ratings were geographically aggregated based on nine PHM regions. Distributions were explored between regions. Multilevel analyses per provider category, which produced Intraclass Correlation Coefficients (ICC), were performed to determine clustering of ratings of providers located within regions. If ratings were clustered, then this would indicate that differences found between regions could be attributed to regional characteristics (e.g. demographics or regional policy).

**Results:**

In the nine regions, 70,889 ratings covering 4100 care providers were available. Overall, average regional scores (range = 8.3–8.6) showed significant albeit small differences. Multilevel analyses indicated little clustering between unsolicited provider ratings within regions, as the regional level ICCs were low (ICC pioneer site < 0.01). At the provider level, all ICCs were above 0.11, which showed that ratings were clustered.

**Conclusions:**

Unsolicited online provider-based ratings are able to discern (small) differences between regions, similar to solicited data. However, these differences could not be attributed to the regional level, making unsolicited ratings not useful for overall regional policy evaluations. At the provider level, ratings can be used by regions to identify under-performing providers within their regions.

## Background

Regional Population health Management (PHM) initiatives are challenged to evaluate regional patient experiences to improve their health and social services. These initiatives have been increasingly widening their focus from individuals to populations [1, 2] to deal with the changing care demand. Their intent is often to achieve the Triple Aim; i.e. simultaneously improve population health and the experienced quality of care, while reducing costs [3]. This, combined with a more general focus in care on how patients’ experience care [4, 5], makes it essential for PHM initiatives to evaluate regional patient experiences.

Many reforms struggle with evaluating population level experienced quality of care to assess their regional policies, resulting in a variety of methods used [6, 7]. Currently, solicited surveys are dominant, with well-known examples such as the Hospital Consumer Assessment of Healthcare Providers and systems (HCAHPS) and the NHS Inpatient Survey [8, 9]. Notwithstanding the value of solicited surveys, they often have substantial downsides such as the significant time lag between measurement and publication, low response rates and high costs to deploy [10, 11]. A potential other source might be found in websites that gather unsolicited online ratings. Analogue to other sectors, where consumers are getting used to voicing their experiences online using for example Yelp.com [12] and other social networks such as Twitter and Facebook [10], patients are increasingly sharing their experiences on the internet [13, 14]. Patients can often rate their care experiences on general websites like Yelp or Facebook, as well as specialized websites such as RateMDs and HealthGrades.

Unsolicited online patient ratings have shown promise for creating insight into experienced quality of care at the provider level. At this level, they seem to be especially useful as an additional perspective or source of information complementing solicited surveys [11, 15, 16] or as a more real-time alternative [17]. In the Netherlands, the most widely used patient rating website is *ZorgkaartNederland.nl* (Dutch Care Map, ZKN) [18], which is run by the Dutch Patient Federation (DPF). Since 2007, over 500,000 experiences with different individual care providers, hospitals and other care institution were shared by patients on the ZKN website. Their experiences might prove valuable for policy makers as it could compile close to real-time information regarding progress on one of the pillars of the Triple Aim and might be of value for comparisons between regions. However, the extent to which these provider level online ratings can be used to create overall insight for (regional) population level policies is unclear. If combined, they could be used to measure overall regional quality of care and aid regional policy evaluations in a relatively simple and cost-effective manner.

Therefore, this study aimed to explore whether unsolicited online provider ratings can be used to create insight into differences in patient experiences between as well as within regions. To asses this, the structure and regional coherence of online unsolicited ratings will be studied. Additionally, a comparison will be made between the results of unsolicited ratings and the dominant method, solicited surveys, in nine regions to explore their differences.

## Methods

### Study population

In 2013 the Dutch minister of Health appointed nine regions as pioneer sites because of their goal to implement regional policies according to the Triple Aim [19]. These pioneer sites are demarcated geographical areas in which different organizations work together to achieve this goal [20]. Each site has their own approach with different organizations involved, such as hospitals, municipalities or insurance companies. They are monitored by the National Institute for Public Health and the Environment in the so-called National Monitor Population management (NMP) and are spread out across the Netherlands. Overall, about 2 million people live in these regions, but the size as well as the characteristics of the population in each region varies (Table 3 in Appendix 1).

### Data sources

Two data sources were used in this study; the primary focus were the unsolicited online patient ratings provided by the DPF, while the solicited survey data provided by the NMP was used predominantly for comparative reasons.

The unsolicited online patient ratings were derived from the www.ZorgkaartNederland.nl (ZKN) website, which was made available by the DPF. On this website, patients can both give and see reviews. To add a review, patients first select a care provider, which can be a care professional like a specific GP or specialist, or an organization such as a hospital (department) or nursing home. Six ratings have to be given, ranging from 1 to 10, covering six quality of care dimensions. The dimensions differ depending on the category of provider that is selected (i.e. for hospital care they are appointments, accommodation, employees, listening, information and treatment). Additionally, there is a textbox where patients can explain their ratings and add other relevant comments as well as the condition they were treated for. No further personal information about the respondent is requested, but a timestamp and email address is registered. The ZKN staff checks each submission for repeated entries, integrality and anomalies, and gives each one an identifier.

The solicited survey data used was provided by the NMP (Ethical Review Board number: EC-2014.39) [21]. In nine pioneer sites, a random sample of 600 insured adults per pioneer site (total = 5400) were invited to fill out the survey between December of 2014 and January of 2015 [21]. This yielded 2491 filled-out surveys (response rate 46.1%), around 300 per pioneer site. The average age was 55.7 years old and more than a quarter was highly educated (Table 3 in Appendix 1). The solicited survey population has previously been described in more detail [22]. For this study only the following question was used: “On a scale from 0 to 10, where 0 is the worst possible care and 10 is the best possible care, which grade would you give the total care you received in the past 12 months?” Thus, in this dataset only ratings given for overall care experiences were available.

### Ratings

In addition to ratings given through the website, the DPF actively gathers ratings by visiting care providers. These visits predominantly focus on residents of nursing homes and each is logged using a unique identifier. To distinguish between ratings given unsolicited and those gathered by the DPF, the number of occurrences of the unique identifier was checked. Identifiers that showed up more than 10 times were labeled as solicited, while others were labeled as unsolicited. A mean rating was calculated for each entry by averaging the six ratings provided. This combination was shown to provide a good summary of an entry [23]. Ratings and providers were clustered at the regional level using the nine pioneer sites’ zip codes [24]. Additionally, a second set of regions was created for the below described sensitivity analyses. These regions were identified using zip codes based on nine regional initiatives that were not included in the NMP [25]. Furthermore, providers in the *Zorgkaart* data were grouped into the following categories: hospital care, nursing homes, general physicians (GP), insurer, birth care, pharmacy, physiotherapy, youth care, dental care and ‘others’. In the survey data, the only alteration made was the combination of the 0 and 1 ratings to create scales that both have 10-points, running from 1 to 10. These ratings were already grouped by pioneer site.

### Analyses

First, descriptive statistics were extracted for both the unsolicited online ratings and solicited survey data to explore the structures of both data sets. Rating frequencies and means were determined per pioneer site overall and for the online data these were also stratified by the largest provider categories; hospital, GPs, dental care and nursing home. To compare the two datasets, means, using independent t-test, and distributions were studied. Additionally, with each dataset an Analysis of Variance (ANOVA) was performed to test for differences between pioneer sites and Spearman’s rho was determined to look at the correlation of mean scores based on both the unsolicited online ratings and solicited survey data.

Second, multilevel analyses were performed using the unsolicited online ratings based on three levels; 1) rating, 2) provider and 3) pioneer site, in order to gain insight in the regional clustering of ratings. If ratings were clustered, then this would indicate that found mean differences between regions could be attributed to characteristics of the regions. The year a rating was given was added as a fixed variable to adjust for changes over time, such as the introduction of population health policies. Using the three levels and the ratings as dependent variables, the model was run to determine intraclass correlation coefficients (ICC, range = 0–1). The ICC is a measure of similarity between values from the same group and provides insight into the clustering of, in this case, unsolicited ratings in regions and in providers. At which level this clustering is meaningful is assessed based on ratio of the between person (i.e. provider) variance and within person variance [26]. To have interpretable results, levels within a multilevel analyses have to be interpretable and similar, therefore models were tested per provider category.

Finally, a sensitivity analysis was added to assure that found results in the multilevel analyses were not due to region selection and would be comparable with other than the nine selected regions. Multilevel analyses were repeated with alternative regions for this purpose.

SPSS 22 (SPSS Inc., Chicago, Illinois) and R Studio Version 0.99.441 for Windows (RStudio, Boston, Massachusetts) were used to perform the analyses described below. A *p*-value below 0.05 was considered significant in all analyses.

## Results

### Ratings

The *Zorgkaart* database provided 449,263 unsolicited ratings, given by 208,047 unique identifiers. Of these unsolicited ratings, 70,800 were given by 31,260 identifiers to providers in the pioneer sites (Table 2 in Appendix 1). Of the 25,616 care providers that received at least a single rating in *Zorgkaart*, 4100 were located in one of the nine pioneer sites (Table 2 in Appendix 1). The total number of ratings varied strongly between pioneer sites, from *n* = 1451 (region *Vitaal Vechtdal*) to *n* = 17,953 (region *SmZ*). However, the population size of the pioneer sites also varied substantially, from 106,270 in *GoedLeven* to 646,910 in *Friesland Voorop.* If expressed in percentages against population size the number ratings varied from 1.3% (region *Vitaal Vechtdal*) to 3.7% (region *PELGRIM*). When further classified by category of care provider, it is shown that dental care, GP care, hospital care, nursing homes and physiotherapy had a substantial number of ratings available per pioneer site. Tables showing the distribution of ratings can be seen in Appendix 1.

**Fig. 1:**
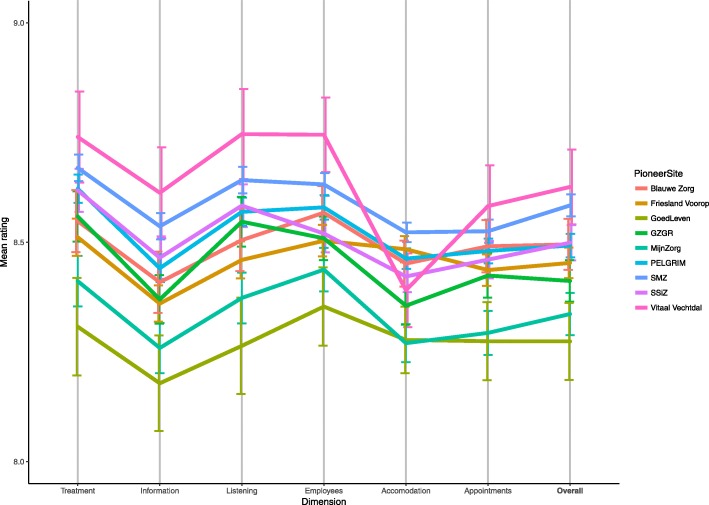
Comparison of mean ratings per dimension and overall per pioneer site with confidence intervals (Range 8–9)

Overall mean scores illustrated that when combining all ratings in a region, differences between regions were significant but small (ANOVA *p* < 0.001). As the limited range of Fig. 1 illustrates, mean unsolicited online ratings of pioneer sites were around 8.5 for each dimension as well as the mean overall scores. When ratings are broken down by care provider category, different patterns emerged. Different pioneer sites stand out, either positively or negatively, in different provider categories (Appendix 2).

When compared to solicited survey ratings, unsolicited online ratings were generally higher (Table 4 in Appendix 1). When individual regions were compared on unsolicited online and solicited survey ratings, all differed significantly in their means (*p* < 0.05). The dispersion of both unsolicited online and solicited survey ratings were both skewed towards the positive, but online ratings slightly more so. For unsolicited online ratings, 8 and 10 were the most dominant ratings, while solicited survey ratings peaked at 7 and 8 out of 10. Comparing mean scores of pioneer sites based on each dataset showed an insignificant correlation (Spearman’s rho = 0.42, *p* = 0.26). Performing relatively well in one dataset, did not mean a region would perform well in the other or vice versa.

### Clustering

Multilevel analyses were only performed for dental care, GPs and nursing homes. These categories had both substantial numbers of ratings as well as rated care providers (Table 2 in Appendix 1). Hospital care had sufficient ratings and individual providers as well, but was excluded as these were mostly given to one or two locations (hospitals) in a region.

The ICCs_region_ from all categories were close to zero indicating there was little clustering between provider ratings within the same site (Table 1). The ICC_providers_ was substantially larger, which indicated that some variance was explained by actual differences between providers. When replacing the third level, pioneer sites, in the sensitivity analysis with the alternate PHM regions, these proportions did not change.

**Table 1:** Intraclass correlation coefficients

Region	ICC	Dental care	GPs	Nursing home
Pioneer sites	ICC_region_	0.002	0.001	0.008
	ICC_providers_	0.154	0.137	0.113
Alternative regions	ICC_region_	0.004	0.003	0.010
	ICC_providers_	0.181	0.151	0.170

*GP* General Practitioner, *ICC* Intraclass Correlation Coefficient

## Discussion

This study explored whether unsolicited online provider based ratings can provide insight into patient experiences at a (regional) population level. The overall mean scores as well mean scores stratified by provider category (e.g. hospital (department) and GP practices) differed significantly between pioneer sites. However, most differences were small as they often only varied a few tenths on a 10-point scale. This in itself is not an issue, as care experiences might be comparable between each region, as similar small differences were found in solicited survey. Unsolicited ratings did overall score higher. Multilevel analyses conducted using the unsolicited online ratings among GPs, dental care and nursing homes at the pioneer site level indicated there was little clustering of ratings between providers in the same region. This makes it difficult to attribute any found variation to regional (i.e. population) level differences. The provider level did show a meaningful clustering of ratings, suggesting differences could be explained by provider variation.

Unsolicited online ratings cannot be used to gain insight in differences between regions for now. There appeared to be little clustering of experienced quality of care between providers in the same region. This lack of regional grouping of experienced quality can also be seen using other measures [27]. Currently, this limits the use of unsolicited online ratings as an evaluation tool for the regional level. Even though the goal was not to evaluate any specific policy, PHM initiatives in the Netherlands have only started implementing regional collaborations five years ago and many are still in the start-up stage. Regional policies have shown the potential to impact quality of care [28], but in the Netherlands, initiatives might require more time to have an impact.

When looking at ratings separated by the individual dimensions, notable patterns emerged. For example, *Vitaal Vechtdal* was rated the highest overall, but had a substantial dip in the accommodation dimension and was rated lowest in the dental care category. Similar interesting patterns could be seen in other pioneer sites and this, keeping the low ICCs in mind, could be insightful for both policy makers and providers. Furthermore, the dispersion in ratings between providers was substantial. This illustrates the variation of experienced quality of care of providers within a region; there are providers that are performing better than other providers. This is in line with previous studies, which showed that care providers in the same region differ in the care experience they deliver [27]. To be able to identify variations in providers is useful for regional policymakers, as it illustrates there is room for poorer scoring providers to be identified and improved. Ideally, by stimulating integration and cooperation within healthcare, overall experienced quality of care should improve and ratings should be more geographically coherent.

This study is the first to explore the use of unsolicited online care provider ratings at the regional level. Results are not yet consistent[16],but several studies show the potential correlation between ratings and hospital readmissions as well as other objective quality of care measures [15, 29]. Furthermore, they can be used by care inspection agencies as an additional input source [18] and have shown to impact real world behavior of consumers [30]. However, online ratings and the used *Zorgkaart* data in particular have limitations that have to be considered. Patients can give more than one rating, making them not completely independent. However, correcting for this using a cross-classified multilevel model, which is not preferred as it is very skewed as most patients give one rating, did not show any different results. Additionally, *Zorgkaarts’* unsolicited ratings are given to providers and adjustments have to be made to be able to evaluate at population level. A more direct measure of general population level experienced quality of care would be preferable. Furthermore, the present number of ratings were insufficient for in-depth analyses for many provider categories in this study (e.g. insurance companies and disabled care). Additionally, several providers had only a few ratings available for the multilevel analysis. The *Zorgkaart* data showed that the frequency at which ratings are submitted has been increasing rapidly over the years and it is therefore expected that the low numbers issue will be solved over time. *Zorgkaart*, as do most rating sites, also has limited participant information for privacy reasons. This means it is impossible to correct for selection bias, while a younger, more tech-savvy population tends to provide ratings [31]. Finally, there was limited opportunity to connect the unsolicited dataset to solicited dataset. The solicited dataset did not target specific quality dimensions like *Zorgkaart*, which prevented a comparison or conclusions at this level. For regions, dimension specific information and comparisons could be useful.

For regional population evaluations, online ratings could be improved. First, it is worth performing a follow-up study in a few years to determine if the same conclusions can be drawn. By this time, more ratings are available and the initiatives will have had more time to form their interventions and have an impact. Second, an algorithm could be created that highlights poor performing or declining providers within a certain area for policy makers to faster identify and aid underperforming providers. Third, text comments accompanying ratings can provide an additional source of information [32] and could provide more detail for policy makers as well as providers [33]. Finally, to truly evaluate regional policies that go beyond healthcare, broader measures are required that cover preventive, well-being and social services. The current ratings are generally only focused on the quality of healthcare services. Expansion of current or the creation of new instruments would be needed to drastically improve their use for current regional health policies that go beyond clinical care.

## Conclusions

The aim of this study was to assess the ability of unsolicited online ratings to provide insight into regional experienced quality of care. Currently, they have limited use for regional evaluations, because even the small differences found could not be attributed to regional characteristics. Providers did show meaningful clustering of ratings, highlighting the ability to identify under-performing providers and opportunities for regional policy.

## Figures and Tables

**Fig. 2:**
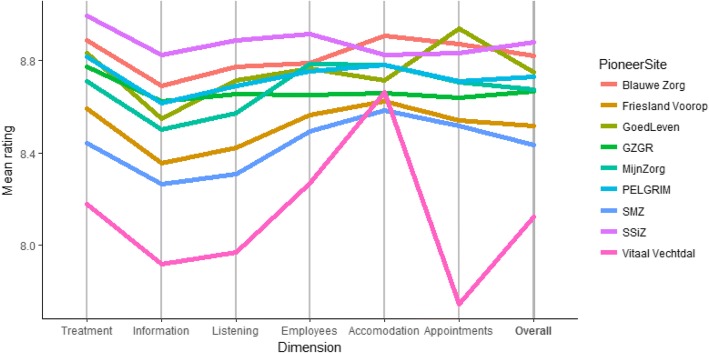
Comparison of mean dental care ratings between pioneer sites (GZGR = Gezonde Zorg, Gezonde Regio; SmZ = Slimmer met Zorg; SSiZ = Samen Sterk in Zorg)

**Fig. 3:**
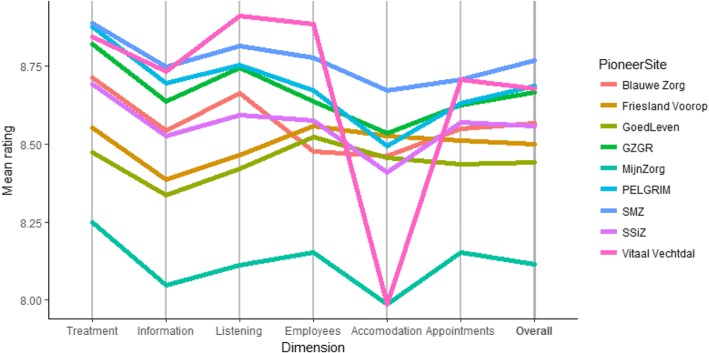
Comparison of mean hospital care ratings between pioneer sites (GZGR = Gezonde Zorg, Gezonde Regio; SmZ = Slimmer met Zorg; SSiZ = Samen Sterk in Zorg)

**Fig. 4:**
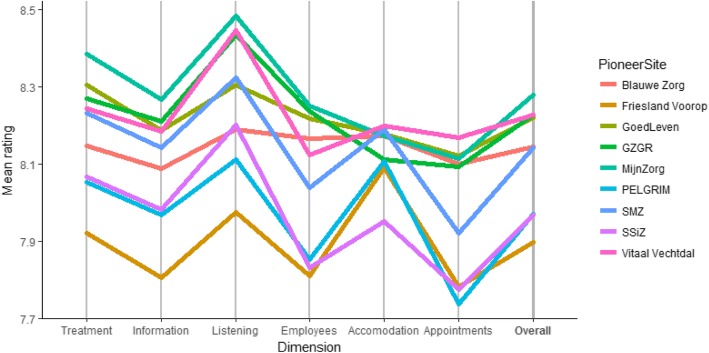
Comparison of mean general practitioner’s ratings between pioneer sites (GZGR = Gezonde Zorg, Gezonde Regio; SmZ = Slimmer met Zorg; SSiZ = Samen Sterk in Zorg)

**Fig. 5:**
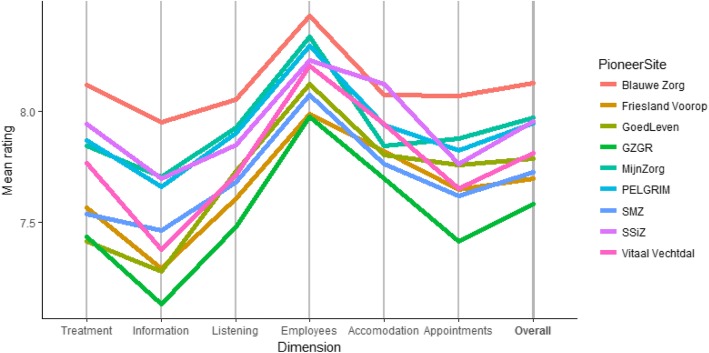
Comparison of mean nursing home ratings between pioneer sites (GZGR = Gezonde Zorg, Gezonde Regio; SmZ = Slimmer met Zorg; SSiZ = Samen Sterk in Zorg)

## Appendix 1

### Descriptions of datasets

**Table 2:** Number of ratings and providers per provider category per pioneer sites

	Blauwe Zorg	Friesland Voorop	GoedLeven	MijnZorg	PELGRIM	GZGR	Smz	SSiZ	Vitaal Vechtdal	Total
	number of ratings/number of providers,									
Birth care	4/3*	174/15	4/1	41/9	626/16	19/3	288/12	16/12	2/2	1174/70
Dental care	364/43	893/121	115/21	819/69	1577/107	667/46	1548/124	952/124	142/20	7077/633
GP care	918/52	1852/181	422/28	1543/73	1603/125	698/55	2590/170	1089/78	202/30	10,917/792
Home care	170/30	131/34	31/7	119/20	540/64	25/8	377/62	72/15	35/10	1500/250
Hospital care	625/2	3150/6	901/4	1178/3	3745/5	1484/3	5645/9	1800/2	503/2	19,031/36
Insurers	0/0	334/2	0/0	38/1	640/2	806/2	0/0	179/1	0/0	1997/8
Nursing homes	323/24	589/89	199/21	787/48	1158/53	290/23	1243/57	454/33	178/22	5221/370
Other	1024/75	3383/165	84/28	1230/97	4168/196	1379/91	4485/239	1868/114	104/31	17,725/1036
Pharmacy	106/17	168/42	44/11	113/25	345/40	77/19	214/55	237/21	7/4	1311/234
Physiotherapy	25,645	609/123	48/21	465/76	869/110	353/43	1556/156	384/66	278/20	4818/660
Youth care	0/0	15/3	0/0	3/1	3/2	0/0	7/4	1/1	0/0	29/11
Total ratings	3790	11,298	1848	6336	15,274	5798	17,953	7052	1451	70,800
Number of citizens	176,055	646,910	106,270	273,500	417,780	183,920	516,500	273,340	112,655	270,6930
Relative number of ratings (%)	2,2	1,7	1,7	2,3	3,7	3,2	3,5	2,6	1,3	–

*GZGR* Gezonde Zorg, Gezonde Regio; *SSiZ* Samen Sterk in Zorg, *SmZ* Slimmer met Zorg

**Table 3:** Descriptives of survey sample population

Population	Blauwe zorg	Friesland Voorop	Goed Leven	MijnZorg	GZGR	PELGRIM	SSiZ	SmZ	Vitaal Vechtdal	ANOVA/Chi^2^	Total study population
Gender (% male)	49.8	43.8	48.5	49.4	47.0	44.4	43.0	45.4	44.3	0.675	46.1
Age (Standard deviation)	57.9 (16.3)	54.3 (16.6)	55.1 (15.8)	59.1 (14.0)	54.5 (17.3)	54.7 (15.3)	59.0 (15.7)	54.7 (16.9)	51.6 (15.1)	0.000	55.7 (16.1)
Education (% highly educated)	34.9	26.7	20.1	18.8	42.4	28.0	22.0	27.1	12.7	0.000	25.8
Origin (% native)	84.1	95.4	80.5	77.9	85.7	84.8	87.5	87.1	93.8	0.000	86.4
Employed (% paid job)	46.9	49.1	48.3	41.2	51.7	50.6	45.2	51.4	63.1	0.000	49.7
Disabled (%)	5.9	3.8	4.2	6.6	5.2	2.6	2.3	5.4	3.1	0.145	4.3
BMI	26.1	25.9	25.9	26.7	25.4	26.4	25.4	25.8	26.0	0.011	26.0
Alcohol use (glasses per week)	3.8	4.7	3.9	3.7	4.6	4.0	5.1	4.6	4.2	0.144	4.3
Smoking (% smokers)	16.5	16.2	20.1	20.1	17.0	14.9	13.7	21.2	23.6	0.048	18.1
Health Literacy (score Chew’s Set of Brief Screening Questions)	3.4	3.5	3.3	3.4	3.4	3.3	3.4	3.3	3.3	0.230	3.4

*GZGR* Gezonde Zorg, Gezonde Regio, *SSiZ* Samen Sterk in Zorg, *SmZ* Slimmer met Zorg

**Table 4:** Comparison and ANOVA of mean scores of online and survey data in nine pioneer sites (*N* = 70,889)

	Online rating (SD)	Survey rating (SD)	Difference
Blauwe Zorg	8.50 (1.82)	7.47 (1.39)	1.02*
Friesland Voorop	8.45 (1.89)	7.82 (1.21)	0.63*
GoedLeven	8.27 (1.92)	7.63 (1.42)	0.64*
MijnZorg	8.34 (1.96)	7.52 (1.50)	0.80*
PELGRIM	8.49 (1.69)	7.69 (1.34)	0.80*
GZGR	8.41 (1.84)	7.59 (1.27)	0.82*
SMZ	8.58 (1.71)	7.73 (1.22)	0.85*
SSiZ	8.50 (1.73)	7.64 (1.28)	0.86*
Vitaal Vechtdal	8.62 (1.65)	7.74 (1.34)	0.88*
ANOVA	0.000	0.000	

**p* < 0.001

## Abbreviations

ANOVA: Analysis of Variance
DPF: Dutch Patient Federation
GP: General physician
GZGR: *Gezonde Zorg, Gezonde Regio*
HCAHPS: Hospital Consumer Assessment of Healthcare Providers and systems
ICC: intraclass correlation coefficients
NMP: National Monitor Population management
PHM: Population health Management
SmZ: *Slimmer met Zorg*
SSiZ: *Samen Sterk in Zorg*
ZKN: *ZorgkaartNederland.nl*
